# Pulmonary mucinous adenocarcinoma: A case report and literature review

**DOI:** 10.1097/MD.0000000000041161

**Published:** 2024-12-27

**Authors:** Minghui Qian, Ruibing Lyu, Long Xiao, Benqi Shi, Nian Liu, Yaqian Yuan, Wenju Wang, Xin Li

**Affiliations:** a Department of Respiratory and Critical Care Medicine, CR&WISCO General Hospital Affiliated to Wuhan University of Science and Technology, Wuhan, Hubei Province, China; b Clinical Medical College, Wuhan University of Science and Technology, Wuhan, Hubei Province, China.

**Keywords:** image representation, pathological diagnosis, pulmonary mucinous adenocarcinoma

## Abstract

**Rationale:**

Pulmonary mucinous adenocarcinoma (PMA) is a rare subtype of lung adenocarcinoma. Computed tomography images of PMA show pneumonia-like findings, solitary pulmonary nodules or ground-glass opacity with consolidation. Misdiagnosis can delay genetic diagnosis. This article reported a case of PMA presenting with unique imaging findings.

**Patient concerns:**

An 85-year-old female patient was admitted to our hospital with persistent-cough and expectoration. Anti-infection treatment was largely ineffective.

**Diagnoses:**

Bronchoscopy with bronchoalveolar lavage and liquid-based cytology revealed the presence of tumor cells. Lung biopsy confirmed the diagnosis of PMA.

**Interventions:**

Symptomatic treatment was administrated, including anti-infection, relief of cough and expectoration. Bronchoscopy and lung puncture were performed to help clarify the cause of the disease.

**Outcomes:**

The patient’s course was uneventful, and she was discharged in good condition. After admission, the patient continued to receive anti-tumor immunotherapy in the oncology department.

**Lessons:**

PMA is a subtype of lung adenocarcinoma and has a low incidence. PMA usually presents with atypical clinical symptoms and signs, and it is difficult to be diagnosed based on imaging findings. It is often undiagnosed and misdiagnosed. Clinicians must increase awareness about the need for timely diagnosis, so as to develop more targeted treatment and achieve a better prognosis.

## 1. Introduction

According to global cancer statistics, lung cancer is the leading cause of cancer-related deaths worldwide, and lung adenocarcinoma as the main subtype, accounts for more than 40% of all lung cancers diagnosed.^[1]^ Adenocarcinoma can also be divided into various subtypes based on tissue morphology, immunophenotype, and genotype, according to the International Association for the Study of Lung Cancer, American Thoracic Society, and European Respiratory Society classification system. Pulmonary mucinous adenocarcinoma (PMA) is a newly recognized and relatively rare type of lung cancer.^[2]^ On computed tomography (CT) PMA typically presents as a solitary nodular shadow or ground-glass opacity with pneumonia-like consolidation, which is easy to cause misdiagnoses and delayed diagnosis.^[3]^ This article reported a case of PMA presenting with unique imaging features.

## 2. Case report

### 2.1. Case information

An 85-year-old female was admitted to our hospital in April 2024 with cough and expectoration for 6 months and aggravated dyspnea for 1 month. The patient is a retired medical worker. In November 2023, the patient developed cough and expectoration (white sputum) without obvious incentives. No signs of discomfort, such as chills, fever, or hemoptysis were observed. Cephalosporins were administrated to relieve cough and expectoration, and symptomatic improvement was observed. One month prior to the admission, the patient experienced more severe cough, expectoration, and dyspnea. Antibiotic treatment was continued, while cough and dyspnea persisted following discontinuation. Physical examination revealed normal vital signs, pulse oxygen saturation of 94% (no supplemental oxygen), and normal lips. Auscultation of, lungs revealed coarse breath sounds with obvious moist rales. Heart and abdominal examination gave negative results. No edema was found in either left or right lower limb. The patient had a history of high blood pressure, coronary heart disease, cholelithiasis and renal cysts, and a history of surgery for benign thyroid nodules. The patient denied a history of diabetes, hepatitis, tuberculosis, smoking, alcohol use, or medication allergies.

### 2.2. Laboratory examination

C-reactive protein and erythrocyte sedimentation rate were elevated. Complete blood count, electrolytes, liver and renal function tests were normal. Tumor marker levels were within the normal range. Both respiratory virus test and viral polymerase chain reaction test yielded negative results. A T-cell spot test for mycobacterium tuberculosis in blood was negative. Sputum smear was positive for epithelial and gram-positive bacteria. Cultivation of fungi in sputum showed filamentous fungi. Acid-fast bacteria and exfoliative cytology were negative in sputum.

### 2.3. Imaging test

**Figure 1.**
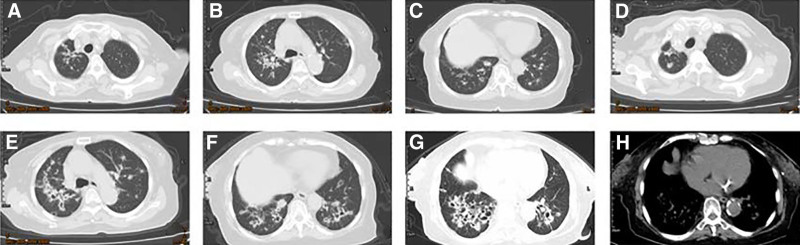
Imaging findings. (A–C) The patient’s early chest CT. (D–F) A chest CT during a period of worsening symptoms despite repeated anti-infection treatment, showing an increase in the lesion size compared to previous scans, with multiple patchy shadows and nodules with cavities in both lungs. (G, H) During hospitalization, after anti-infection treatment, the chest enhanced CT showed no significant change. CT = computed tomography.

Lung CT in November 2023 showed bilateral bronchiectasis with infectious changes and calcification (proliferative and fibrous) in the right upper lobe (Fig. 1A–C). Lung CT in March 2024 showed bilateral thickening of the pulmonary vasculature, with blurred edges; trabs, plaque-shaped high-density shadows, and nodular calcified density shadows in the right upper lobe; multiple plaque-shaped, blurred-margin shadows and nodules in both lungs, and partially visible cavities. The lesion was enlarged as compared to that observed in November 2023 (Fig. 1D–F).

### 2.4. Treatment

**Figure 2.**
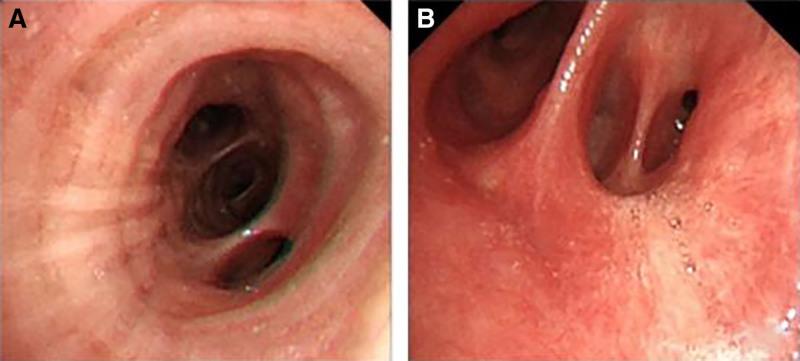
Bronchoscopy. The bronchial mucosa was smooth, and the lumen was patent without deformation or narrowing. No ulcers or neoplasms were seen. (A) Left lower lobe bronchial lumen. (B) Right lower lobe bronchial lumen.

**Figure 3.**
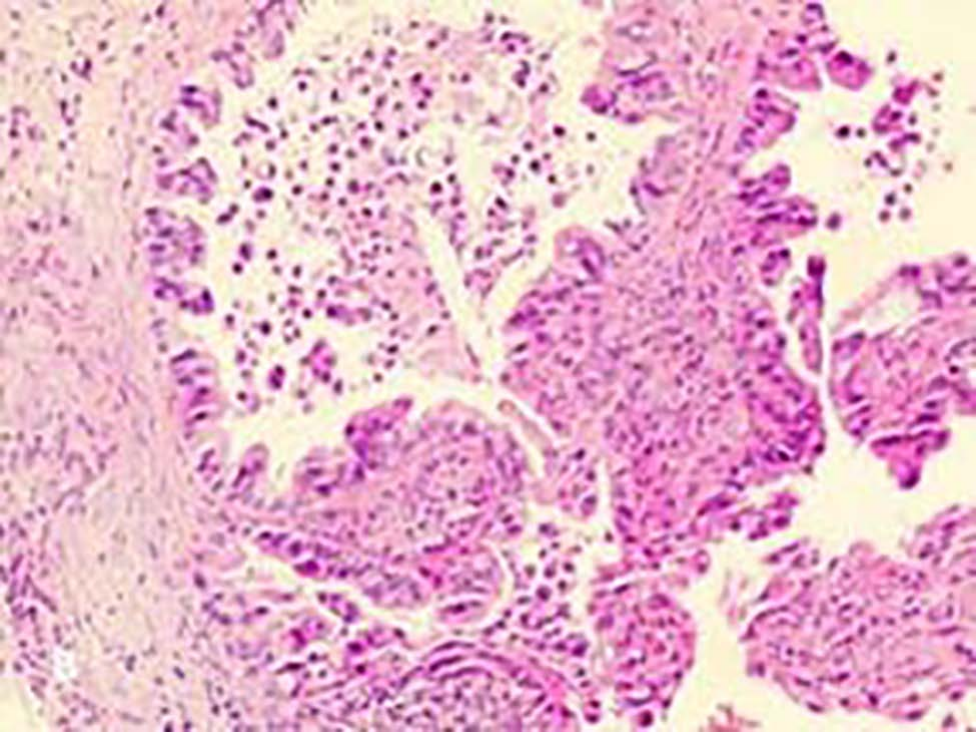
Microscopic appearance of needle biopsy. A biopsy stained for pulmonary mucinous adenocarcinoma (H&E × 200), goblet cells, and columnar cells was visible, with abundant mucus in the cell cytoplasm and substantial mucus secretion outside the cells.

Given the presence of pulmonary infection, anti-infection was obtained by use of Azithromycin aspartate. In the meantime, Doxofylline was prescribed for relief of bronchospasm, Ambroxol and Acetylcysteine Capsules plus Montelukast Sodium Tablets were given to resolve cough and expectoration, and Ipratropium bromide was administrated for nebulization. However, cough and dyspnea persisted. Bronchoscopy was performed on the third day after admission (Fig. 2). Bronchoalveolar lavage fluid (BALF) was culture-negative for bacteria and, fungi. Hairbrush-derived bacteria, anti-acid bacteria, and Mycobacterium tuberculosis were negative. BALF gram stain revealed leukocytes, epithelia, and a small number of gram-positive cocci. Liquid-based cytology analysis revealed abundant heterocysts with large nuclei under the microscope, and some heterocysts showed an adenoid structure. BALF metagenomic next generation sequencing suggested Streptococcus constellatus and Aspergillus fumigatus. No obvious changes were observed on contrast-enhanced chest CT on March 2 and April 12 (Fig. 1G, H). CT guided percutaneous lung biopsy revealed PMA in the right lower lobe (Fig. 3). Immunohistochemistry showed caudal-related homeobox transcription factor 2 (−), recombinant cytokeratin 7 (CK7; +), recombinant Ki-67 protein: 20% to 30% (+), Napsin A (−), tumor suppressor protein, oncogene protein (strong diffusion+), thyroid transcription factor 1 (−), and Villin (+). PMA (cT4N1M1 IV) was diagnosed, and surgery was not considered suitable. No gene mutation/fusion was found in epidermal growth factor receptor (EGFR), Kirsten rat sarcoma viral oncogene homolog (KRAS), anaplastic lymphoma kinase, or ROS proto-oncogene 1, receptor tyrosine kinase. Tislelizumab based immunotherapy was given on May 4, 2024, and mild toxicity developed. The patient is now in good general condition and is still under follow-up.

## 3. Discussion

PMA is a rare subtype of lung adenocarcinoma, accounting for only 2% to 10% of all adenocarcinomas diagnosed.^[4]^ Symptoms of PMA mainly include cough and expectoration.

PMA has diverse CT features that can generally be divided into 2 types: nodular and pneumonia-like types. Nodular lesions usually present as short and tiny lesions with spiculation sign, lobulation sign, vacuole sign, and pleural traction sign. Most of the lesions are distributed around the lungs, and a small number of the lesions lie in the central parts of the lungs. They have attenuation values similar to water but lower than muscle. Pneumonia like lesions exhibit imaging findings similar to pneumonia, such as lamellar consolidation accompanied by ground-glass density opacity. In addition, air bronchogram and leafless tree signs can be observed.^[3]^ In the study of Watanabe et al^[5]^ 75% of PMA patients were reported with solitary pulmonary nodules or lumps, and the other 25% exhibited a pneumonia like pattern. In the case reported here, the chest CT findings appeared atypical. Multiple plaque-shaped blur shadows and cavity-accompanied nodules were observed, making it difficult to differentiate from pulmonary infectious diseases and then leading to misdiagnosis at early stages. Moreover, the diffuse, patchy, and blur shadows that were presented were not specific, as they can be found in various pulmonary infectious diseases such as pneumonia and pulmonary tuberculosis. Research^[6]^ shows that diffuse and patchy shadows are rarely presented in PMA cases, while patchy consolidation in both lungs are relatively common. Pulmonary cavitary lesions are shared by various diseases, including tuberculosis, non-tuberculous mycobacterial infection, and constitutional or metastatic lung cancer. As reported,^[7]^ only 7% to 13% of PMA cases present with cavitary lesions. In addition, PMA patients with cavitary lesions tend to have a worse prognosis than patients without a cavitary lesion. In this study, the initial diagnosis of PMA could not be confirmed radiographically, due to certain heterogeneous imaging findings BALF analysis showed a small amount of mucus and shedding tumor cells. Combined with percutaneous lung biopsy result, the diagnosis of PMA was confirmed. This case report may help guide future diagnosis of PMA. However, more cases are needed, and followed-up data are required to help us better understand this disease. It is believed that PMA will be increasingly recognized with the development of science, which will help optimize the diagnosis and treatment of this disease.

The diagnosis of PMA relies on histopathological tests, which is similar to other lung adenocarcinomas. PMA has the pathological features of cotyloid or columnar tumor cells with abundant mucus in the cytoplasm, karyons at the fundus, small volume, and lower heteromorphism than non-mucinous adenocarcinoma.^[8]^ The positivity rates of PMA’s main immunologic markers are approximately 90% for CK7, 50% for recombinant cytokeratin 20, and 9% for caudal-related homeobox transcription factor 2.^[9]^ In the case reported here, the patient presented with pathological features in accordance with PMA, and CK7 immunohistochemical stain showed a positive result.

The mutation rate in PMA driver genes is low, and the predominant gene mutations in PMA have been summarized in several studies. KRAS mutations are the most common mutations in PMA, accounting for 35% to 75% of cases.^[10,11]^ The mutation rate of tumor suppressor protein, oncogene protein in PMA is approximately 46%. Other common gene mutations include EGFR, anaplastic lymphoma kinase, and ROS proto-oncogene 1, receptor tyrosine kinase gene mutations, and the mutation rate of EGFR gene is only 4.5%.^[12]^ In this report, no driver gene mutations were detected. This is consistent with previous studies. Surgery and chemotherapy are primary treatment approaches for PMA. For patients who harbor gene mutations, cannot tolerate surgery and have a poor response to chemotherapy, targeted therapy will be performed based on the mutation sites. In recent years, immunotherapy represented by immune checkpoint inhibitors has been continuously developed. Immune checkpoint inhibitors targeting programmed cell death protein 1/programmed cell death-ligand 1 have become a new standard for treatment of advanced small cell lung cancers.^[13]^ PD-L1 expression is very low in mucinous adenocarcinomas. Nakagomi et al^[14]^ reported a rate of 6.1% and 59.7% of PD-L1 expression in invasive mucinous and non-mucinous adenocarcinomas, respectively. Here, the patient was old and diagnosed with IV PMA (cT4N1M1). Neither surgery nor chemotherapy was considered. The patient was tested negative for mutations in KRAS and EGFR gene. Immunophenotyping of PD-L1 was untested. Tislelizumab was given and exhibited a good curative effect.

## 4. Conclusion

PMA is a rare subtype of lung adenocarcinoma, and has a low incidence rate. It usually presents with atypical clinical symptoms and signs, and it is difficult to be diagnosed based on imaging finding. Thus, PMA is often undiagnosed and misdiagnosed. The diagnosis of PMA depends on histopathological and immunohistochemical tests. For pneumonia patients who fail to respond to antibiotic treatment, percutaneous lung biopsy or tracheoscopy should be performed early to obtain a pathological diagnosis and buy more time for treatment.

## Author contributions

**Conceptualization:** Xin Li.

**Resources:** Benqi Shi, Nian Liu, Yaqian Yuan.

**Supervision:** Wenju Wang.

**Writing – original draft:** Minghui Qian, Long Xiao.

**Writing – review & editing:** Ruibing Lyu, Xin Li.
